# *Aggregatibacter actinomycetemcomitans* H-NS promotes biofilm formation and alters protein dynamics of other species within a polymicrobial oral biofilm

**DOI:** 10.1038/s41522-018-0055-4

**Published:** 2018-05-22

**Authors:** Kai Bao, Nagihan Bostanci, Thomas Thurnheer, Jonas Grossmann, Witold E. Wolski, Bernard Thay, Georgios N. Belibasakis, Jan Oscarsson

**Affiliations:** 1 Division of Oral Diseases, Department of Dental Medicine, Karolinska Institutet, Huddinge, Solnavägen, Sweden; 2 Division of Oral Microbiology and Immunology, Center of Dental Medicine, University of Zürich, Zürich, Switzerland; 3 Functional Genomics Center, ETH Zürich and University of Zürich, Zürich, Switzerland; 4 Oral Microbiology, Department of Odontology, Umeå University, Umeå, Sweden

## Abstract

*Aggregatibacter actinomycetemcomitans* is a Gram-negative organism, strongly associated with aggressive forms of periodontitis. An important virulence property of *A. actinomycetemcomitans* is its ability to form tenacious biofilms that can attach to abiotic as well as biotic surfaces. The histone-like (H-NS) family of nucleoid-structuring proteins act as transcriptional silencers in many Gram-negative bacteria. To evaluate the role of H-NS in *A. actinomycetemcomitans*, *hns* mutant derivatives of serotype a strain D7S were generated. Characteristics of the *hns* mutant phenotype included shorter and fewer pili, and substantially lower monospecies biofilm formation relative to the wild type. Furthermore, the D7S *hns* mutant exhibited significantly reduced growth within a seven-species oral biofilm model. However, no apparent difference was observed regarding the numbers and proportions of the remaining six species regardless of being co-cultivated with D7S *hns* or its parental strain. Proteomics analysis of the strains grown in monocultures confirmed the role of H-NS as a repressor of gene expression in *A. actinomycetemcomitans*. Interestingly, proteomics analysis of the multispecies biofilms indicated that the *A. actinomycetemcomitans* wild type and *hns* mutant imposed different regulatory effects on the pattern of protein expression in the other species, i.e., mainly *Streptococcus* spp., *Fusobacterium nucleatum*, and *Veillonella dispar*. Gene ontology analysis revealed that a large portion of the differentially regulated proteins was related to translational activity. Taken together, our data suggest that, apart from being a negative regulator of protein expression in *A. actinomycetemcomitans*, H-NS promotes biofilm formation and may be an important factor for survival of this species within a multispecies biofilm.

## Periodontal disease: Protein promoting biofilm formation

A member of a specific group of gene-regulating proteins promotes biofilm formation by a bacterium associated with aggressive forms of gum disease. Forming biofilms helps the bacterium to cause persistent infections. Researchers at Karolinska Institutet and Umeå University (Sweden), and University of Zürich (Switzerland), led by Jan Oscarsson at Umeå University, investigated the role of the “histone-like” protein H-NS in *Aggregatibacter actinomycetemcomitans* infections. These proteins are known to suppress the activity of specific genes in many bacteria, a property confirmed in this research. By studying mutant bacterial strains deficient in H-NS protein, the researchers demonstrated that this protein promotes the formation of biofilms by the bacteria. The results suggest that H-NS plays a significant role in allowing *Aggregatibacter actinomycetemcomitans* to thrive in biofilms containing mixed populations of bacteria. This effect appears to involve activating production of hair-like appendages called pili on the bacterial surface.

## Introduction

*Aggregatibacter actinomycetemcomitans* is a Gram-negative bacterium from the *Pasteurellaceae* family. Colonization by *A. actinomycetemcomitans* is strongly associated with aggressive forms of periodontitis in adolescents and young adults.^1^ The microorganism is also a pathogen, associated with non-oral infections, such as endocarditis,^2^ and is a candidate bacterial trigger of anti-citrulline autoimmunity in rheumatoid arthritis.^3^ Some of the virulence factors of *A. actinomycetemcomitans* involved in oral colonization and induction of periodontal inflammation have been thoroughly studied and well characterized, including, e.g., leukotoxin (LtxA) and cytolethal distending toxin (for a recent review see ref.^4^). An important virulence property of *A. actinomycetemcomitans* is its ability to form tenacious biofilms that can attach to abiotic as well as biotic surfaces.^5^ Adherence and biofilm growth in *A. actinomycetemcomitans* is mediated by the tight-adherence (*tad*) gene locus, which consists of 14 genes (*flp-1*, *flp-2*, *tadV*, *rcpCAB*, *tadZABCDEFG*),^6,7^ and also depends on the production of an extracellular carbohydrate polymer of β (1,6)-linked *N*-acetyl-d-glucosamine.^8^

The histone-like (H-NS) family of DNA-binding, nucleoid-structuring proteins is widespread in Gram-negative bacteria.^9^ H-NS appears to act primarily as a global silencer of AT-rich DNA acquired by horizontal gene transfer,^10^ but evidence has also been presented that it stimulates translation of genes with suboptimal ribosome-binding sequences.^11^ H-NS has been demonstrated to globally control the expression of ~5% of all genes in *Escherichia coli*, many of which are involved in transcription and translation and in the production of cell envelope components required for adjustment to varying environments.^12^ However, in other species, the number of genes regulated by H-NS appears to be more restricted.^9^ H-NS plays a central role in the regulation of virulence-associated genes in several pathogens, and *hns* mutants have been demonstrated to exhibit reduced virulence in various in vitro and in vivo models.^13,14^ For example, in *Actinobacillus pleuropneumoniae* and *Vibrio cholerae*, H-NS acts as a repressor of exopolysaccharide biosynthesis genes, suppressing biofilm formation.^15,16^ On the other hand, in *E. coli*, deletion of the *hns* gene decreased biofilm formation,^17^ whereas accordingly it was increased upon expression of the *hns* gene in trans.^18^

*A. actinomycetemcomitans* encodes an H-NS protein, which is 132 amino acids, and exhibits ~50% amino acid identity to the 15.5 kDa *E. coli* protein. The H-NS protein of *A. actinomycetemcomitans* is well conserved among strains, i.e., identical proteins are encoded by all 38 genome-sequenced strains available in the National Center for Biotechnology (NCBI) database, representing all serotypes (a–g). To date, the function of H-NS in *A. actinomycetemcomitans* has not been described. Hence, we sought to investigate if H-NS may act as a global regulator in *A. actinomycetemcomitans*, and to examine the influence of H-NS on biofilm formation. It was recently demonstrated that presence of *A. actinomycetemcomitans* in an in vitro multispecies oral biofilm mimicking subgingival dental plaque had a regulatory effect on the other species, i.e., their overall protein expression profiles were altered.^19^ This prompted us in the present work to assess whether an *A. actinomycetemcomitans* wild type and *hns* mutant might induce different patterns of altered protein expression in the multispecies oral biofilm.

## Results and discussion

### Inactivation of the *A. actinomycetemcomitans hns* gene results in impaired biofilm growth in monospecies biofilm

**Fig. 1:**
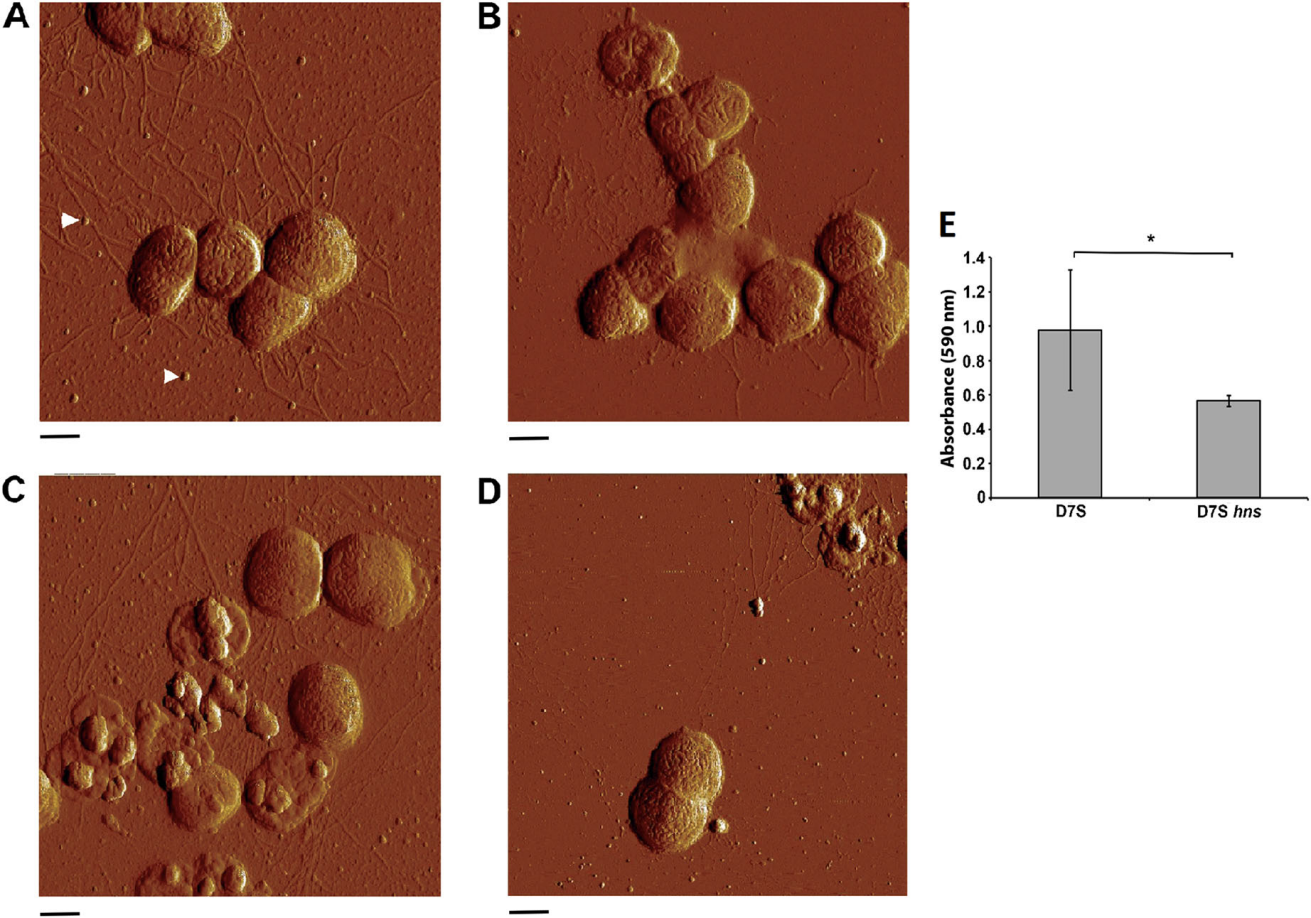
Assessment of the influence of H-NS on *A. actinomycetemcomitans* cell morphology and monoculture biofilm formation. Atomic force microscopy was used to analyze the following *A. actinomycetemcomitans* strains, cultivated on agar at the indicated temperatures: D7S 30 °C (**a**), D7S *hns* 30 °C (**b**), D7S 37 °C (**c**), and D7S *hns* 37 °C (**d**). Arrows indicate examples of the released OMVs. Scale bars = 500 nm. Crystal violet was used to quantify the biofilm formation of the strains D7S and D7S *hns*, respectively, after cultivation in 24-well cell culture plates for 3 days at 37 °C (**e**). Shown are the means of OD 590 nm ± standard deviation (SD) for six experiments; *p *=* 0.0169 for D7S vs. *D7S hns*

To investigate if lack of *hns* expression in *A. actinomycetemcomitans* might result in apparent phenotypical differences regarding gene expression with emphasis on virulence, *hns* mutants were generated in strains D7S and D7SS, as described in the Methods section. The abolished H-NS production in the mutants was confirmed using western blot, and a polyclonal antiserum made against *E. coli* H-NS (Supplementary Fig. 1). To investigate if inactivation of *hns* caused a visible effect on the cell morphology, atomic force microscopy (AFM) was conducted, assessing strains D7S and D7SS, and their corresponding *hns* mutants, cultivated at 30 and 37 °C, respectively. According to our results (Fig. 1a–d), at both temperatures the D7S *hns* mutant appeared less piliated, exhibiting pili that were shorter than those of the parental strain. However, no apparent dissimilarity was seen comparing the smooth-colony type derivative, D7SS, and its *hns* mutant (data not shown). Moreover, in contrast to findings with *E. coli*,^20^ AFM revealed no substantial difference in the amount of outer membrane vesicles (OMVs) released by the wild type and the *hns* mutant in either D7S or D7SS (Fig. 1a–d, and data not shown). To test whether the reduced piliation in the D7S *hns* mutant might be associated with impaired biofilm growth, monocultures of the strains cultivated at 37 °C were stained with crystal violet (Fig. 1e). This revealed decreased (reduced to approximately 58%; *p* < 0.05) biofilm formation in the D7S *hns* mutant relative to the wild type. Together, these observations support the notion that *hns* may act as an activator of pili production in *A. actinomycetemcomitans*, promoting biofilm growth.

### Inactivation of the *hns* gene results in impaired growth of *A. actinomycetemcomitans* in a multispecies oral biofilm, without altering the proportions of the other species

**Fig. 2:**
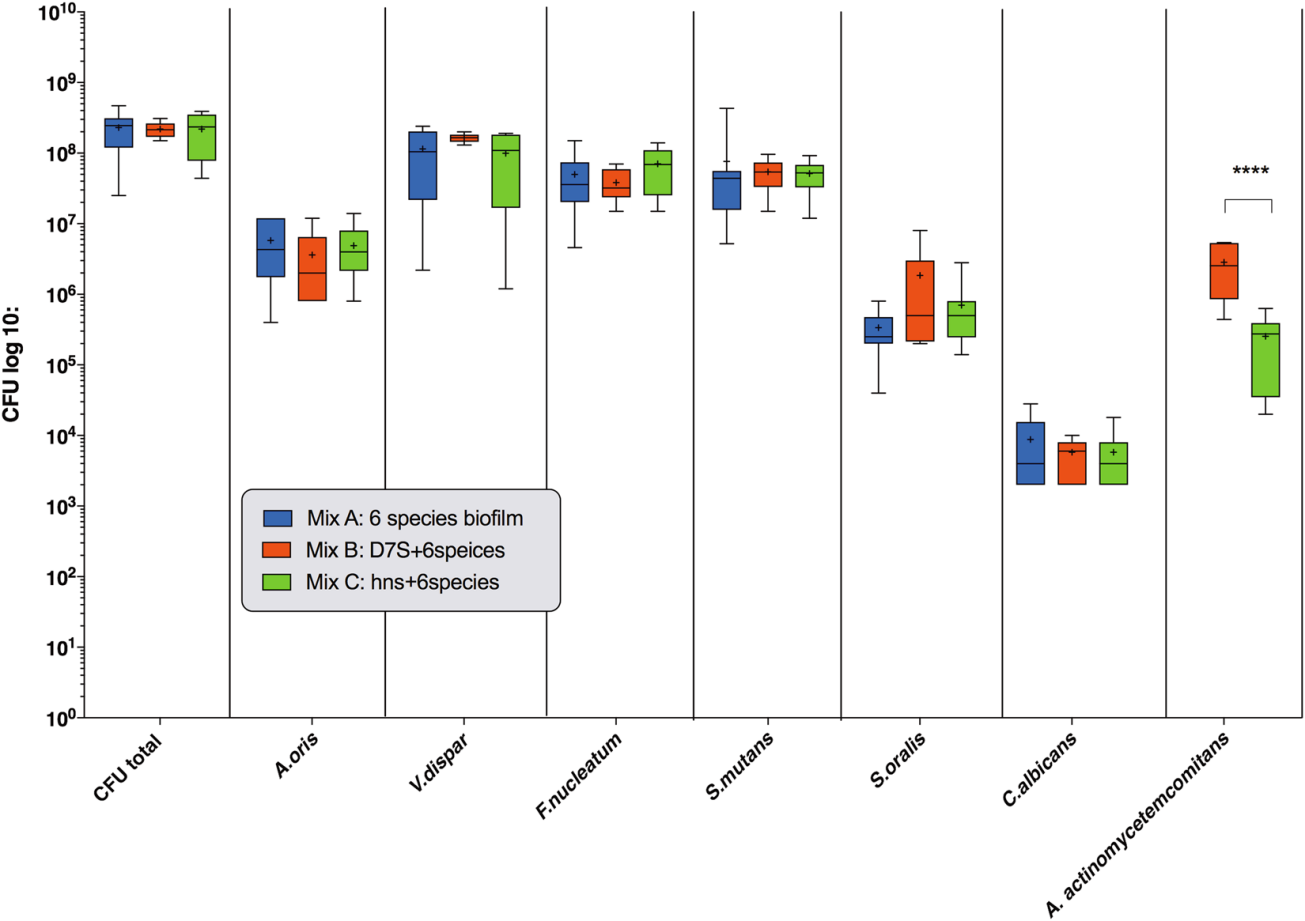
Analysis of the quantitative composition of the multispecies oral biofilms. Quantification was performed used CFU counting for each species per biofilm disc as described in the Methods. As indicated, control biofilms did not include an *A. actinomycetemcomitans* strain (Mix A), whereas experimental biofilms either contained *strain* D7S (mix B) or its *hns* mutant (Mix C). The data are expressed as the bacterial mean counts ± SD from 10 biological replicates. ANOVA test: ****p < 0.0001

We next investigated the influence of the *hns* mutation on the growth in a multispecies (*n* = 6) biofilm setting. After anaerobic cultivation for 64 h at 37 °C, the numbers of the individual oral microbial species within the biofilms were estimated by colony-forming unit (CFU) counting (Fig. 2). Comparative investigation of strain D7S and its *hns* mutant within the biofilm context, respectively, revealed that there were significantly lower numbers of the *hns* mutant, i.e., more than 11 times reduced relative to the wild type (*p* < 0.0001) (Fig. 2). This is consistent with the reduced biofilm formation of the *hns* mutant observed in monocultures (Fig. 1). On the other hand, neither D7S nor D7S *hns* had a significant impact on the growth of the other six species within the biofilm, as compared to when the biofilm was cultivated without an *A. actinomycetemcomitans* strain (Fig. 2).

### Inactivation of the *A. actinomycetemcomitans hns* gene does not alter the structure of the multispecies oral biofilm

A further investigation of the structure of the multispecies biofilm by means of confocal laser scanning microscopy (CLSM) corroborated the CFU results. This revealed the localization of the *A. actinomycetemcomitans* cells along with the other species. Evidently, strain D7S cells appeared to be more abundant in the biofilms as compared to cells of the D7S *hns* mutant (Supplementary Fig. 2). On the other hand, CLSM revealed no apparent alterations regarding the general structural conformation of the whole biofilms.

### Inactivation of the *hns* gene alters the proteome of *A. actinomycetemcomitans* strain D7S

**Fig. 3:**
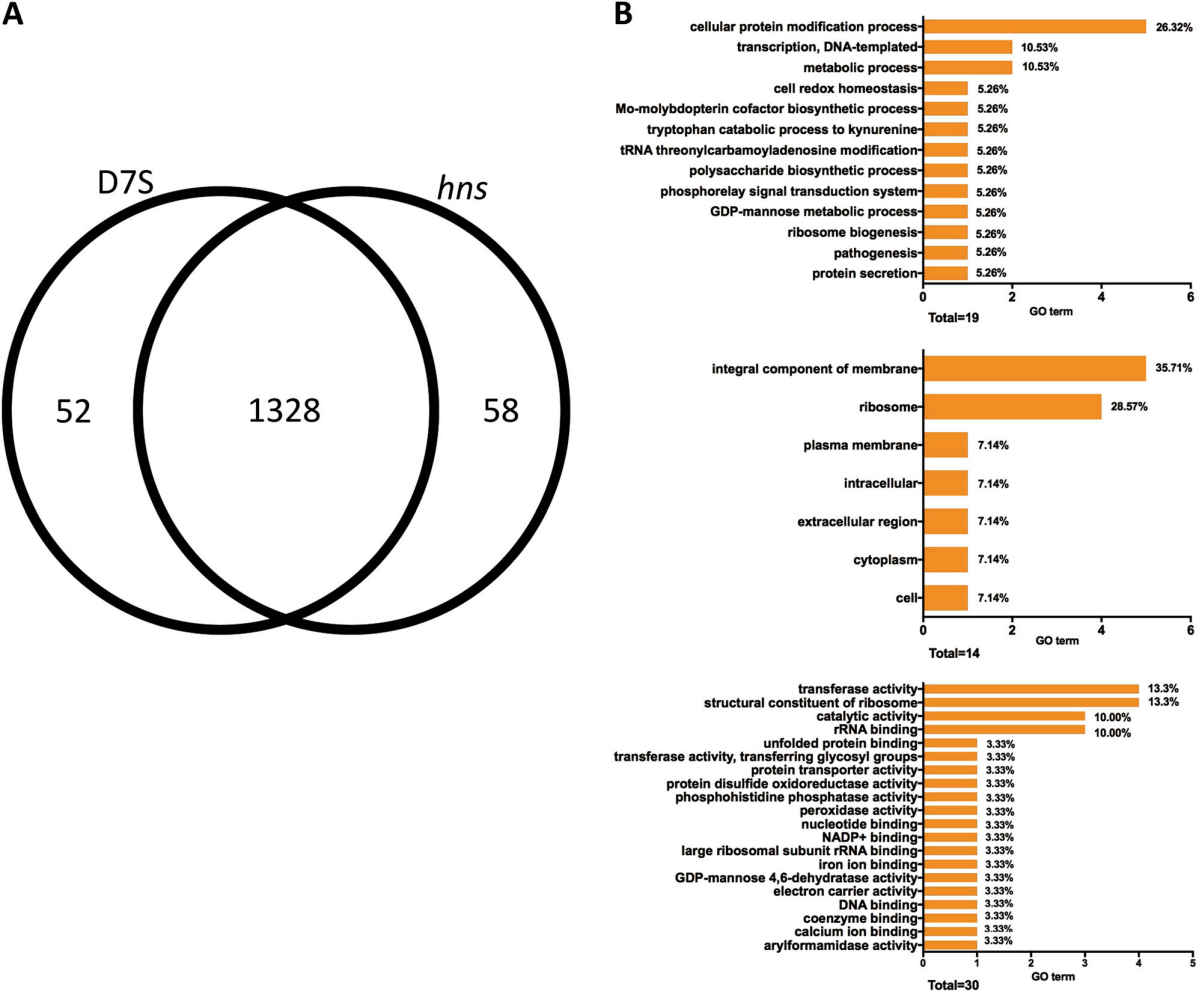
H-NS-dependent proteome of *A. actinomycetemcomitans* strain D7S. Venn diagram indicating the numbers of identified proteins in *A. actinomycetemcomitans* strain D7S and D7S *hns* monocultures grown on agar (**a**). Gene ontology (GO) analysis of the 29 proteins found to be repressed by H-NS (**b**). The numbers and proportions of GO terms from each of the three categories, biological process, cellular component, and molecular function, are shown. Sub-domains with their GO proportion less than 2% were classified as “other”

To investigate if H-NS may act as a regulator of multiple genes in *A. actinomycetemcomitans*, we investigated the influence of the *hns* mutation on the whole proteome of strain D7S. For this, whole cell lysates from the agar monocultures were collected and processed for quantitative proteomics analysis. For each strain, three independent protein preparations were analyzed. For identification, proteins with at least two unique peptides were accepted. A total of 1438 proteins with corresponding false discovery rate (FDR) of 3% at the protein level and 0.18% at the peptide level were identified (Supplementary Table 1). The majority of proteins were identical in both variants (1328 out of 1438). In all, 58 proteins were exclusive to the wild type D7S, whereas 52 were found only in the *hns* mutant strain (Fig. 3a). Quantitative differences in protein expression were assessed by the Progenesis software, as detailed in the Methods section. Heat maps were used to assess the correlation between samples (Supplementary Fig. 3A). It was found that the D7S and D7S *hns* samples were clustered in two separate groups. The quantification by the Progenesis software showed that 31 proteins were differently expressed among these two strains with analysis of variance (ANOVA) *p* value <0.05 and log_2_ fold ≥1 (Supplementary Table 2). Thus, under these conditions, the H-NS-regulated proteome constituted a relatively small proportion of the protein-encoding genes (i.e., 2581 in D7S).^21^ The majority of these 31 proteins, i.e. 29, were upregulated in the *hns* mutant, which is consistent with the notion that H-NS is mainly acting as a repressor of gene expression in *A. actinomycetemcomitans*. The two proteins that showed significantly higher levels in D7S relative to the *hns* mutant strain were HK1651_07880 (i.e., H-NS), and ABC transporter ATP-binding protein.

To determine putative cellular functions repressed by H-NS, the 29 proteins were grouped according to their Gene Ontology (GO) terms for their predicted biological process, cellular component (i.e., subcellular localization), and molecular function, respectively (Fig. 3b). A majority of these proteins were integral components of membrane (35.71%) or structural constituent of ribosome (28.57%), which is in line with findings in other species, e.g., *E. coli*.^12^ Concomitantly, the two most predominant molecular functions of these proteins were structural constituent of ribosome (13.33%) and transferase activity (13.33%). Regarding the biological processes repressed by H-NS, the majority of the proteins were represented in cellular protein modification processes (26.32%), metabolic process (10.53%), and transcription, DNA-templated (10.53%). Of the known virulence factors, we observed that two proteins encoded by the leukotoxin (*ltxCABD*) gene locus, i.e., leukotoxin (LtxA), and LtxD, were among the proteins upregulated in the *hns* mutant. This is consistent with findings with the homologous hemolysin operons *hlyCABD* and *ehxCABD* in *E. coli*, and *rtxACBD* in *V. cholerae*, which are also repressed by H-NS.^13,22,23^ Functional homologs to some of the other upregulated proteins in D7S *hns* have been shown earlier to promote virulence, including biofilm formation in other species, e.g., glycosyl transferases,^24^ galactose metabolism pathway proteins,^25^ and prepilin peptidase.^26^ Moreover, TorR (also known as ArcA) is a two-component system response regulator and a global regulator of virulence factor expression.^27^ Albeit implied by AFM (Fig. 1a–d), we detected no significant regulation of any component encoded by the *tad* gene locus. For Flp-1, the major fimbrial subunit, this may be a result of low detection efficiency in liquid chromatography– tandem mass spectrometry (LC-MS/MS) due to its small molecular size (8 kDa) or to limited sensitivity for low abundant proteins. Our data also identified several unknown proteins repressed by H-NS, which might represent factors affecting virulence and biofilm development. Among these, the protein exhibiting the highest level of upregulation in the D7S *hns* mutant versus the wild type was hypothetical protein ACT75_01010. Whether they may play a role in *A. actinomycetemcomitans* virulence and biofilm formation will be subject to future studies.

### *A. actinomycetemcomitans* D7S and D7S *hns* induce different patterns of protein expression in the microbial species of the oral biofilm

**Fig. 4:**
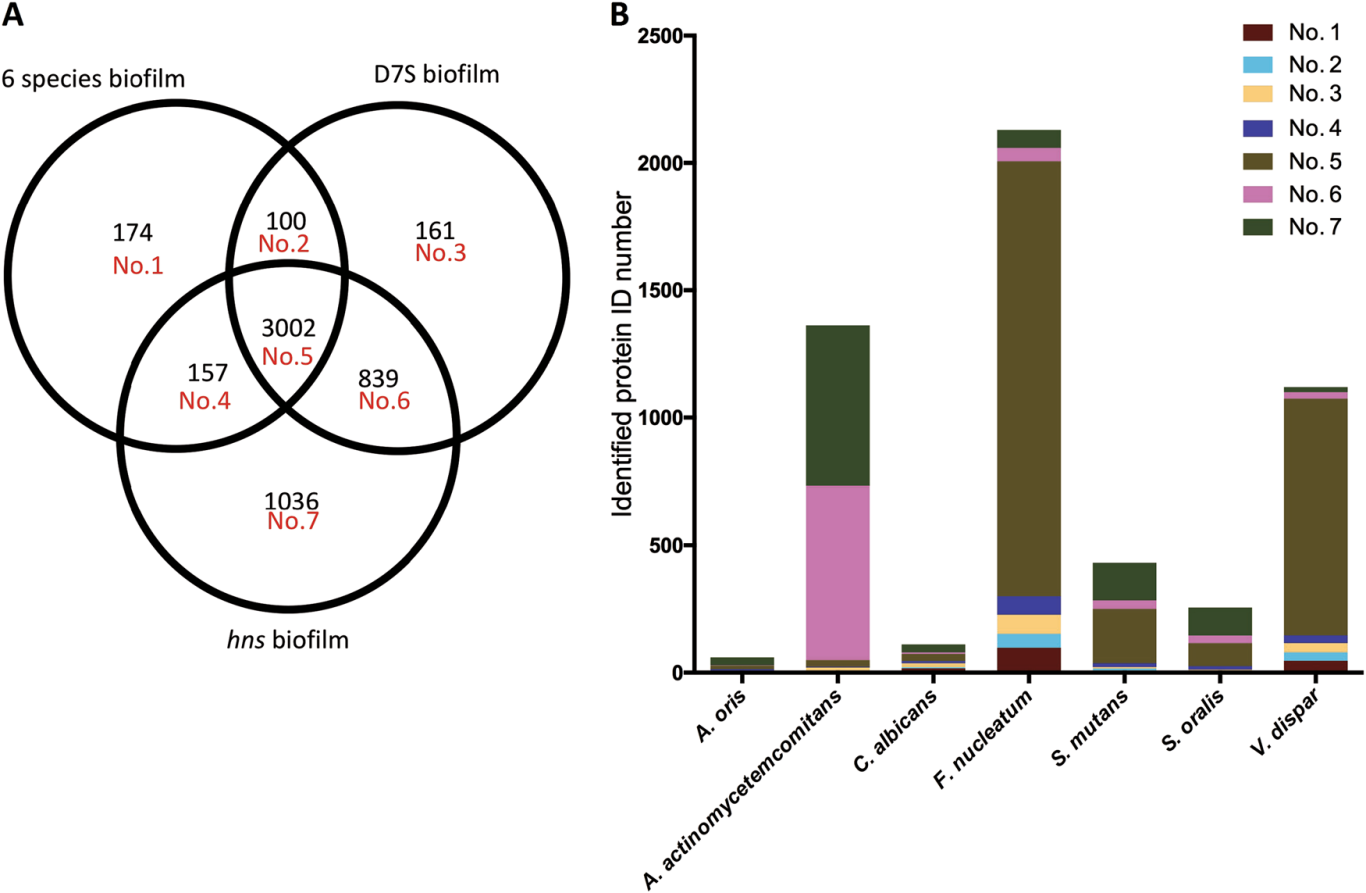
Qualitative analysis of the differential effect of *A. actinomycetemcomitans* strain D7S and D7S *hns* on protein expression in a multispecies oral biofilm. Venn diagram indicating the numbers of identified proteins in the three different biofilms using LC-MS/MS (**a**). Numbers of identified proteins per species in the biofilms are indicated in the bar chart (**b**) with corresponding domain numbers from the Venn diagram

To evaluate if D7S and D7S *hns* may differentially induce proteomic changes in multispecies oral biofilms, lysates from the D7S (*n* = 10), the D7S *hns* (*n* = 10), and the control biofilm (*A. actinomycetemcomitans* excluded; *n* = 9) were collected and processed for proteomics analysis. From these analyses, a total of 5469 proteins with FDR of 2.8% on protein level and 0.68% on peptide level were identified (Supplementary Table 3). Comparisons of the numbers of identified proteins in the different biofilms and for each species in the biofilms are shown in Fig. 4a, b, respectively. According to our data, 3002 proteins were identified in all three biofilms, and a relatively large number of the 1036 proteins were uniquely identified in the D7S *hns* biofilm. Moreover, there was an overlap of 839 proteins between the D7S and D7S *hns* biofilms. For *F. nucleatum* and *V. dispar*, most of their identified proteins (1706 of 2130 *F. nucleatum* proteins, and 927 of 1120 *V. dispar* proteins) were identified in all three biofilms, whereas for *A. actinomycetemcomitans* (627 of 1362 proteins), *S. mutans* (147 of 431 proteins), and *S. oralis* (109 of 225 proteins), a relatively large fraction of the proteins was merely identified in the D7S *hns* biofilm.

**Fig. 5:**
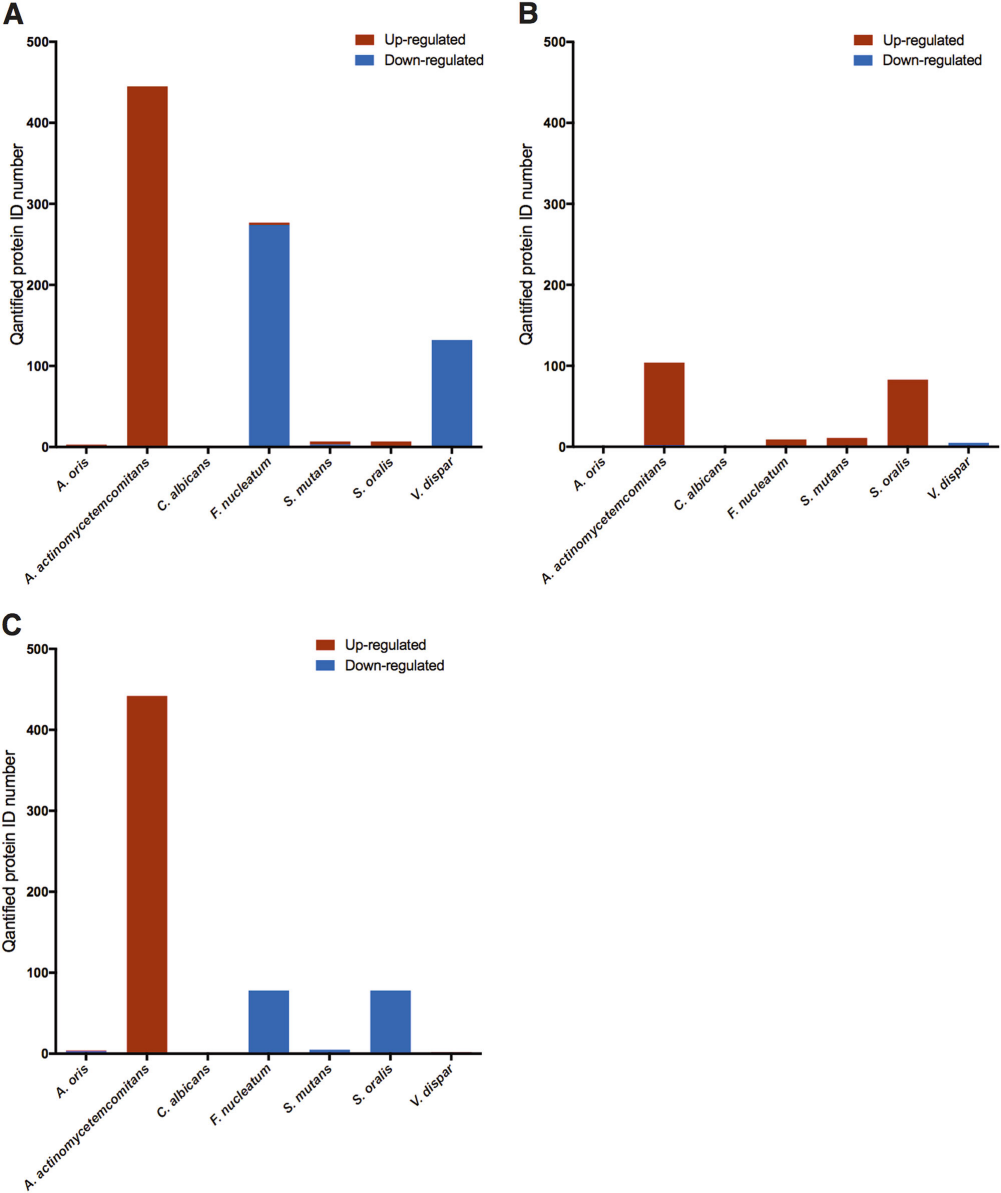
Quantitative analysis of the differential effect of *A. actinomycetemcomitans* strain D7S and D7S *hns* on protein expression in a multispecies oral biofilm. Regulation trends of label-free quantified proteins from each species are shown in the figure based on whether they were upregulated (brown) or downregulated (blue) in **a** D7S compared with 6 species biofilms, **b** D7S *hns* compared with 6 species biofilms, and **c** D7S compared with D7S *hns* biofilms

According to the label-free quantification results, the presence of an *A. actinomycetemcomitans* strain resulted in altered protein expression in the multispecies biofilm compared to when it was absent. Interestingly, when biofilms were clustered based on the overall protein expressions, D7S biofilms formed one group, whereas the D7S *hns* and the 6-species control biofilms (control biofilm without D7S) were clustered together in another group based on our algorithm (Supplementary Fig. 3). Considering the tendency of Progenesis to aggressively match features,^28^ *A. actinomycetemcomitans* proteins were not included in these analyses when comparisons were made against the control, i.e., either of the *A. actinomycetemcomitans*-containing biofilms versus the 6-species biofilm. The total numbers of proteins identified as regulated between the different biofilms were 872 in the D7S wild type versus the control biofilm without D7S (Supplementary Table 4), 214 in the D7S *hns* versus the control biofilm without D7S (Supplementary Table 5), and 610 in the D7S versus the D7S *hns* biofilm (Supplementary Table 6). Comparing the pattern of regulated non-*A. actinomycetemcomitans* (non-*Aa*) proteins in the D7S versus the 6-species control biofilm (Fig. 5a) revealed that 16 were upregulated in the D7S biofilm, whereas a large majority, i.e., 411, were downregulated. The downregulated proteins mainly originated from *F. nucleatum* (*n* = 274) and *V. dispar* (*n* = 132). In contrast, when comparing the D7S *hns* with the control biofilm without D7S (Fig. 5b), the total number of regulated non-*Aa* proteins was much lower, and the majority (*n* = 104) were up- rather than downregulated (*n* = 8). These regulated proteins mainly originated from *Streptococcus* spp. (11 *S. mutans* and 83 *S. oralis* proteins, all being upregulated), *F. nucleatum* (8 upregulated and 1 downregulated), and *V. dispar* (5, all downregulated) in the D7S relative to the 6-species biofilm.

Our results are consistent with the notion that the *A. actinomycetemcomitans* strain D7S and its *hns* mutant promote distinctly different proteomic changes in the multispecies oral biofilms, i.e., as judged by the numbers of enhanced protein expression, the regulatory impact on the other biofilm species appeared to be largely compromised by the *hns* deletion. For example, the relatively large downregulation of proteins in *F. nucleatum* and *V. dispar* was observed only when D7S was present in the multispecies biofilm, whereas D7S *hns* instead caused a significant alteration of proteome (i.e., upregulation) in *S. oralis*.

For the species exhibiting different patterns of expressed proteins when co-incubated with D7S and D7S *hns*, respectively, several potential relationships with *A. actinomycetemcomitans* have been described. These include evidence that *F. nucleatum* co-aggregates with *A. actinomycetemcomitans* among a large number of other oral microorganisms.^29^ Results obtained with multispecies biofilm models suggest a potential mutualistic relationship, i.e., that *A. actinomycetemcomitans* and *F. nucleatum* may support the growth of each other in the oral cavity.^30^ Moreover, *F. nucleatum* was found to enhance the attachment and invasion of *A. actinomycetemcomitans* to epithelial cells.^31^ As early colonizers of the oral biofilm, *Streptococcus* spp. were shown to localize on the tooth surface, optimizing the microenvironment for later colonizers.^32^ Autoinducer-2, a member of a family of signaling molecules for quorum sensing, is expressed by both *Streptococcus* spp.^33^ and *A. actinomycetemcomitans*,^34^ suggesting that interspecies signaling might occur between *A. actinomycetemcomitans* and *S. oralis*. In addition, *A. actinomycetemcomitans* can digest lactate secreted by *Streptococcus* spp.^35^ However, in spite of these potential relationships, very few *S. oralis* proteins were regulated in the biofilm when the *A. actinomycetemcomitans* wild-type strain was present. This is consistent with our previous findings with the multispecies biofilm model using an *hns*^+^ *A. actinomycetemcomitans* strain,^19^ suggesting that the regulatory effect on *S. oralis* is mediated by factors normally suppressed by H-NS. The large amount of *S. oralis* proteins that were uniquely identified (Fig. 4b) is also consistent with this hypothesis. Finally, comparing the D7S with the D7S *hns* biofilm, we observed that all 442 regulated *A. actinomycetemcomitans* proteins exhibited higher levels in the D7S biofilm (Fig. 5c). This, which would argue against H-NS acting as a repressor in the multispecies biofilm, may be at least partly a result of the impaired growth (i.e., lower abundance) of D7S *hns* in the biofilm (Figs. 1b and 2). In contrast, *F. nucleatum* proteins (*n* = 78) and *S. oralis* (*n* = 78) constituted a large majority of the 164 that were downregulated in the D7S versus the D7S *hns* biofilm.

### Different patterns of biological pathways expressed in the multispecies biofilm containing D7S and D7S *hns*, respectively

**Fig. 6:**
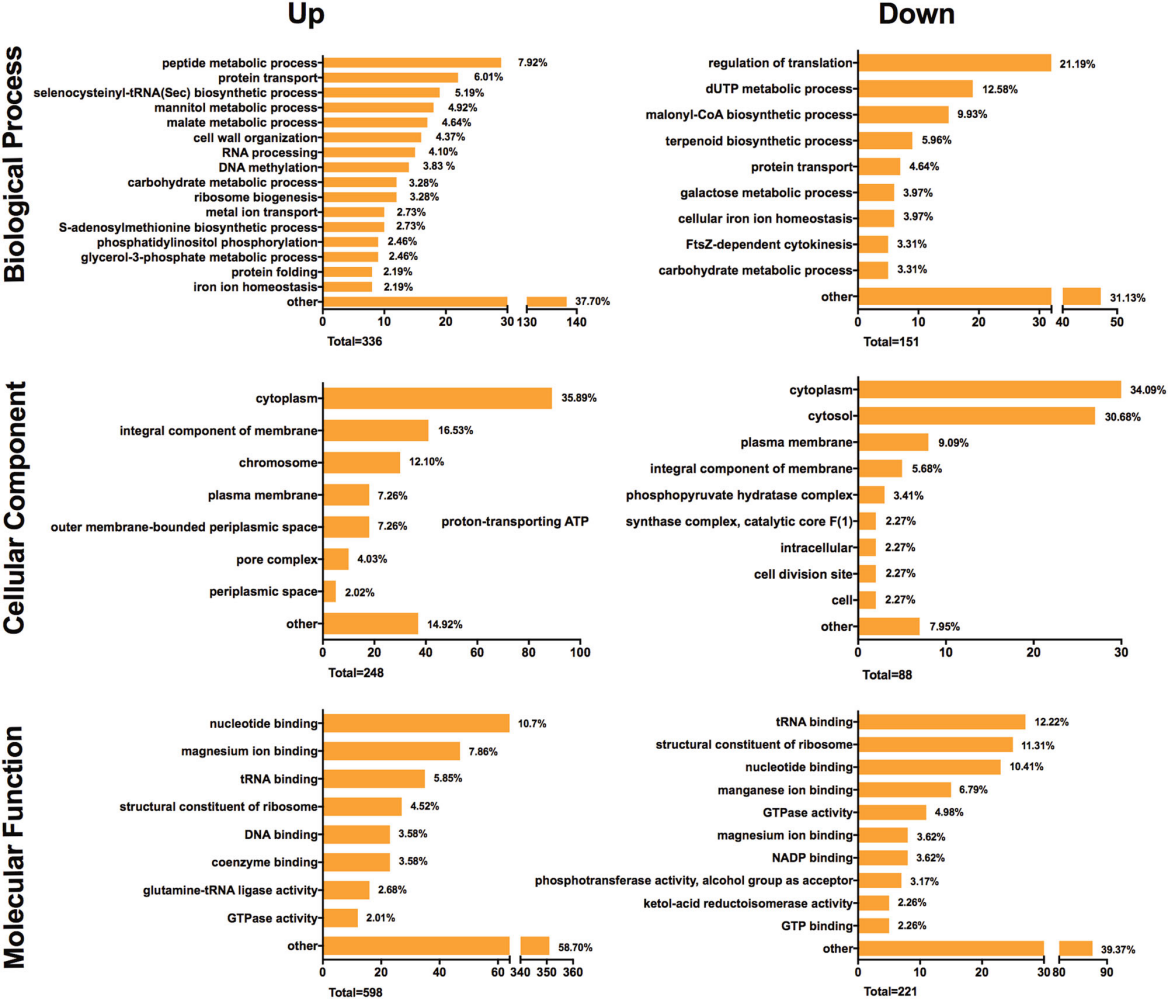
Functional classification of proteins differentially regulated in the multispecies oral biofilms containing *A. actinomycetemcomitans* strain D7S and D7S *hns*, respectively. All up- or downregulated bacterial protein functions in the D7S relative to the D7S *hns* biofilms were annotated by enrichment of Gene Ontology (GO) terms, and displayed as proportions (%) of the total numbers of regulated (up/down) GO terms. Indicated is the numbers of GO terms for biological process, cellular component, and molecular function, respectively. Sub-domains with their GO proportion less than 2% were classified as “other”

As D7S and D7S *hns* differentially induced proteomic changes in the species of the multispecies biofilm, we aimed to assess an overview of the potential functional differences of the protein profiles expressed in the two biofilms (i.e., the D7S versus the D7S *hns* biofilm). To this end, all individual proteins, including also *A. actinomycetemcomitans* proteins, were grouped according to their GO terms for their predicted biological process, cellular component (i.e., subcellular localization), and molecular function, respectively. The GO terms of all the regulated proteins were collectively pooled to decipher the patterns of biological pathways expressed in the multispecies biofilm containing either strain D7S or D7S *hns* (Fig. 6). In brief, 366, 248, and 598 GO terms from the biological process, cellular component, and molecular function category, respectively, were generated from the proteins that were upregulated in the D7S biofilm. The enriched GO terms from the downregulated proteins were 151, 88, and 221 from these three domains. In general, both the up- and downregulated proteins in the D7S versus the D7S *hns* biofilm have diverse functions with 31.13 and 58.70% GO terms enriched in the “other” category in biological process and molecular function domains. This is similar to observations with *E. coli* that H-NS represses a large number of poorly characterized, unrelated genes.^12^ Interestingly, the proteins expressed at higher levels in the D7S *hns* biofilm contained relatively large proportions of GO terms associated with the biological process “regulation of translation” (21.19%), and with the molecular functions “tRNA binding” (12.22%) and “structural constituent of ribosome” (11.31%). Thus, our data suggest that mutational loss of *A. actinomycetemcomitans* H-NS had a significant impact on the overall translational activity of the biofilm. This would be in accordance with observations that H-NS controls multiple genes involved in transcription and translation in Gram-negative bacteria,^12^ albeit putative indirect mechanism(s) behind the regulatory effects onto the other species remain to be characterized. As cell–cell physical interactions via pili can moderate bacterial swarming behavior,^36^ it cannot be excluded that pili could be involved in inter-bacterial interactions, impacting on regulatory pathways controlling protein expression.

## Concluding remarks

In the present work we have used quantitative proteomics to investigate the role of H-NS in gene expression in the *A. actinomycetemcomitans* model strain D7S, cultured in both mono- and multispecies biofilm settings. We employed the “supragingival” variant of the in vitro multispecies biofilm model, as in the clinical settlement *A. actinomycetemcomitans* is more likely to establish first in supragingival plaque, before expanding subgingivally. We demonstrated that H-NS acted as a repressor of gene expression in *A. actinomyctemcomitans*, which is consistent with its role in several other Gram-negative bacteria. Our present study also underscores the importance of this global gene regulator for the behavior of this organism in a multiple species bacterial community. It will be of interest to elucidate the mechanism(s) of how H-NS promotes *A. actinomycetemcomitans* biofilm formation and contributes to the survival of this species within the multispecies biofilm, which was not revealed from our present proteomics data. Our results are consistent with the notion that H-NS caused qualitative and quantitative proteomic alterations in the multispecies biofilm, and therefore likely contributed to the ecological pressure exerted by *A. actinomycetemcomitans* onto the other species. Although GO terms associated with translational activity were highly abundant among the proteins upregulated in the *hns* mutant multispecies biofilm, we also concluded that there was a large amount of H-NS-regulated proteins corresponding to various unrelated functions (i.e., GO terms classified as “other”). These poorly characterized genes may frequently have been acquired by horizontal gene transfer,^37^ and it cannot be excluded that H-NS may play a role in silencing such genes in *A. actinomycetemcomitans*, and/or in facilitating their sequence diversification as has been demonstrated in other bacteria.^38^

## Methods

### Bacterial strains and growth conditions

*A. actinomycetemcomitans* wild-type fimbriated strain D7S (serotype a) was originally isolated from a patient with aggressive periodontal disease, and D7SS is a smooth colony derivative of D7S.^39^ Mutant derivatives of D7S and D7SS with allelic replacement of the *hns* gene, i.e., D7S *hns*::*kan* [Kan^r^] and D7SS *hns*::*kan* [Kan^r^], were generated in the present work. *A. actinomycetemcomitans* strains were routinely cultivated in air supplemented with 5% CO_2_ at 37 °C for 3 days unless otherwise stated. For this we used blood agar plates (5% defibrinated horse blood, 5 mg hemin/l, 10 mg Vitamin K/l, Columbia agar base), on tryptic soy agar plates or tryptic soy broth (TSB), supplemented with 0.6% yeast extract, and 0.8% glucose (Difco). Alternatively, for transformation assays, the strains were grown on trypticase soy broth supplemented with 0.1% yeast extract, 5% heat-inactivated horse serum, and 1.5% agar (sTSB agar), and when needed, supplemented with 100 μg/ml (final concentration) kanamycin. For biofilm growth of *A. actinomycetemcomitans* strains, 2 × 10^8^ bacterial cells were inoculated in 2 ml tryptic soy broth (Difco) in 24-well cell culture plates (Nunc), which were incubated in static culture in air supplemented with 5% CO_2_, at 37 °C for 3 days. Biofilms were stained with crystal violet as previously described^40^ and the amount of bound dye, which is proportional to the biofilm mass, was quantitated by measuring its absorbance at 590 nm. *Escherichia coli* laboratory strain DH5α^41^ was used for maintenance of plasmids, and *E. coli* strains JGJ102 (*hns*^+^) and JGJ103 (*hns*)^42^ were used as controls in western blot. *E. coli* strains were cultured aerobically at 37 °C in Luria-Bertani (LB) broth or on LB broth solidified with 1.5% (w/v) agar.

### Generation of strain D7S and D7SS *hns* allelic replacement mutants

A PCR-based approach following standard cloning procedures^43^ was used to construct *hns* gene replacement mutants in *A. actinomycetemcomitans* strains D7SS and D7S. Strain D7S-1 complete genome (GenBank accession CP003496) was used as reference in oligonucleotide synthesis. In brief, PCR fragments flanking the gene locus encoding the H-NS protein (GenBank AFI86019) were amplified using primers H1 (5’-CGCCTTGTAGAAAATCCACGCC-3’) with H2 (5’-CAATCCAAGCAGAATTCGATAAAGGTAAG-3’), and H3 (5’-TACGCAAGCTACGAATTCTCGTTAATATT-3’) with H4 (5’-CTTACACCACCGGTGACTAAAGATAC-3’). The PCR primers H2 and H3 introduced an *Eco*RI restriction site (underlined sequences), allowing ligation of the PCR fragments to flank the kanamycin resistance gene from pUC4K.^44^ Ligation products were then used to transform D7SS on agar plates using procedures described earlier.^39^ The *hns*::Kan allele of D7SS *hns* was transferred to D7S, using natural transformation,^39^ generating D7S *hns*. Confirmation of allelic replacements and the orientation of the inserted resistance cassette were done by DNA sequencing and PCR. For this we used primer H1 and H4, respectively, in combination with a primer specific for the kanamycin determinant (H7: 5’-GATTTATTCAACAAAGCCGCCGTCC-3’).

### Western blot

Standard procedures for sodium dodecyl sulfate–polyacrylamide gel electrophoresis and western blot analysis were used.^43^ For immune detection, we used a rabbit polyclonal antiserum specific for *E. coli* H-NS^45^ (final dilution 1:10,000). As secondary antibody, anti-rabbit horseradish peroxidase-conjugate was used (Jackson ImmunoResearch, Newmarket, UK) (1:10,000). Immunoreactive bands were visualized using Clarity™ Western ECL Substrate (Bio-Rad) and the ChemiDoc™ XRS+ System (Bio-Rad).

### Atomic force microscopy

For AFM, bacterial cells were diluted with ultrapure water (Millipore) and placed onto a freshly cleaved mica surface. Samples were incubated for 5 min at room temperature, washed with ultrapure water, and then placed in a desiccator for ~2 h in order to dry. The samples were finally magnified through a Nanoscope V Atomic Force Microscope (Bruker AXS GmbH, Karlsruhe, Germany), using tapping mode. Final images were plane fitted in both the *x-* and *y*-axes and are presented in amplitude mode.

### Multispecies biofilm formation and harvesting

In addition to *A. actinomycetemcomitans*, the following six oral microbial strains were used in this study: *Actinomyces oris* (OMZ 745), *Candida albicans* (OMZ 110), *Fusobacterium nucleatum* subsp. *nucleatum KP-F2* (OMZ 598), *Streptococcus oralis SK248* (OMZ 607), *Streptococcus mutans UA159* (OMZ 918), and *Veillonella dispar ATCC 17748T* (OMZ 493). A multiple-species biofilm with the latter 6 species (*A. actinomycetemcomitans* excluded) was cultivated as previously reported,^46^ and is in the present work referred to as “control biofilm” in experiments including *A. actinomycetemcomitans*. Two modified 7-species biofilms, namely the D7S biofilm and the D7S *hns-*deficient biofilm, were also developed in parallel. Briefly, 200 μl of each species with similar densities (OD550 = 1.0 ± 0.05) were loaded on the hydroxyapatite dishes and anaerobically incubated for 64 h. During the incubation, the cultivated medium was replenished at 16 h and 40 h. The biofilm dishes were dip-washed in 0.9% w/v NaCl at 16 h, 20 h, 40 h, 44 h, 48 h, and 64 h. After being developed, biofilms were then either suspended in 0.9% w/v NaCl for CFU count as well as proteomic analysis, or fixed in 4% paraformaldehyde for image analysis.

### Image analysis with CLSM

The paraformaldehyde-fixed biofilms were stained by fluorescence in situ hybridization and subjected to CLSM for imagine analysis. For this we used Act639, cy3-labeled 16S rRNA oligonucleotide probe of *A*. *actinomycetemcomitans* (5’-CTCCAGACCCCCAGTATG-3’; formamide concentration: 40%, and NaCl concentration in wash buffer: 46 mM)^47^ and FUS664, cy5-labeled 16S rRNA oligonucleotide probe of *F. nucleatum* (5’-CTTGTAGTTCCG C/T ACCTC-3’; formamide concentration: 40%, NaCl concentration in wash buffer: 46 mM).^48^ YoPro-1 iodide and Sytox Green (1:1 v/v) (Thermo Fisher) were used to counterstain the biofilm following the protocol previously reported.^49^ All images were captured with a 63× objective (glycerol immersion, NA 1.3, Leica Microsystems) on a Leica sp5 confocal microscope (Leica Microsystems). The filters on microscope were set to 500–540 nm, 570–630 nm, and 660–710 for the detection of colors from YoPro-1 iodide and Sytox Green mixture, Cy3 and Cy5, respectively. The captured images were processed using Imaris software (version 7.4.0, Bitplane) to reconstruct the biofilm.

### CFU count on selective plates

The CFU counts were performed to quantify the numbers of individual species in different biofilm models. Biofilm suspensions were diluted into seven 10-fold serial dilutions in 0.9% w/v NaCl to obtain at least one plate containing 20–200 CFUs. Briefly, 50 μl of each diluted suspension was plated using a EDDY Jet Auto Spiral Diluter (IUL instruments). Difco™ mitis salivarius agar plates (Becton, Dickinson and Company) supplemented with 0.001% w/v sodium tellurite (BDH Chemicals Ltd) were used to select *S. oralis* and *S. mutans*. Fastidious anaerobe agar plates (Neogen) containing 1 mg/l erythromycin (Sigma-Aldrich), 4 mg/l vancomycin (Sigma-Aldrich), and 1 mg/l norfloxacin were used to selectively grow *F. nucleatum*. Biggy agar plates (Difco) were used to selectively grow *C. albicans*. Columbia blood agar plates (Oxoid) supplemented with 5% whole human blood were used to selectively cultivate the remaining species. Ten biological replicates were performed for counting purposes.

### Bacterial and biofilm protein extraction

Bacterial lysates for LC-MS/MS were obtained as follows. Multispecies biofilm pellets for control (*n* = 9), D7S wild-type (*n* = 10) and D7S *hns mutant* (*n* = 10) were collected and lysed from suspensions as previously described.^19^ Three biological replicates of *A. actinomycetemcomitans* strain D7S and *A. actinomycetemcomitans* strain D7S *hns* grown on blood agar as monospecies biofilm were also processed using the same method. Briefly, sample preparations were randomized and the samples were suspended with 30 μl lysis buffer consisting 4% w/v sodium dodecyl sulfate (SDS), 0.1 mM dithiothreitol, and 100 mM Tris-HCl pH 8.2, heat shocked at 95 °C for 5 min, and then sonicated (UTR2000, Hielscher) 3× 3 min with 0.5 cycle for intervals at 65 % ultrasonic amplitude. The protein concentrations of lysed mixtures were evaluated using Qubit Protein Assay Kit (Life Technologies).

### Filter-aided sample preparation digestion and C18 clean-up

Microcon YM-30 centrifugal filter unit (Millipore) was used to lyse the samples and remove SDS contamination following the protocol described previously.^19^ Briefly, 200 μl of urea buffer containing 8 M urea, 0.1 mM dithiothreitol, and 100 mM Tris/HCl buffer (pH 8.2) were mixed with 20 μg of each lysed sample and loaded in a filter unite. Each of these mixtures was denatured with additional 200 μl of urea buffer, alkylated with 100 μl of 0.05 M iodoacetamide, and washed three times with 100 μl 0.5 M NaCl. Reagents were then completely removed by centrifugation at 14,000 × *g* for 20 min (or 17 min, for removal of NaCl) at 35 °C. The samples were digested overnight by trypsin (Sigma-Aldrich) in enzyme/protein ratio = 1:50 w/w at room temperature and desalted with StageTips, C18 material, 200 µl tip (Thermo Scientific). The samples were then concentrated using a Speedvac (Thermo Savant SPD121P, Thermo Scientific) and stored at –20 °C until further use.

### LC-MS/MS analysis

Each sample was divided into two technical replicates. The desalted samples were reconstituted with 3% acetonitrile in 0.1% formic acid, and a pooled sample of all samples was prepared to serve as an alignment reference in the quantification analyses stage. Randomization for sample run order was applied and the samples were individually analyzed in a Orbitrap Fusion mass spectrometer (Thermo Fisher Scientific), coupled with EASY-Spray™ LC Columns (Thermo Scientific) column and emitter for chromatographic separation, and a linear gradient of acetonitrile/water (2 to 35% acetonitrile in 80 min, containing 0.1% formic acid) with a flow rate of 300 nl/min, and an automatic switching between MS and MS/MS scans using a top-12 method. MS spectra were acquired for a mass range of 300–1700 *m/z* in profile mode at a resolution of 60,000 at *m/z* 400. The high collusion-induced dissociation fragmentation was performed on 28 normalized collision energy at high resolution.

### Protein identification and label-free quantification

All raw files from LC-MS/MS were searched with Mascot (version 2.5.1) against a database containing 1,535,919 sequences; 551,265,940 residues of human, bacterial, and fungal proteins including those from *A. oris*, *Aggregatibacter aphrophilus*, *A. actinomycetemcomitans*, *C. albicans*, *F. nucleatum*, *S. mutans*, *Streptococcus oralis*, and *V. dispar*. All sequences were downloaded from NCBI on 27 May 2016, and concatenated to 261 sequences known as MS contaminants and reversed (decoyed) to generate the search database. The following parameters were set for the database search: tryptic digests, max two missed cleavages for each peptide, iodoacetamide derivative as a fixed modification on cysteine, acetylation on the protein N-term, deamidation on asparagine to glutamine, and oxidation on methionine residues as variable modification. Peptide tolerance was set to ±10 ppm, and MS/MS tolerance to ±0.6 Da. Mascot search results were imported into Scaffold (version 4.2.1, Proteome software) for validation of the MS/MS-based peptide and protein identifications. The following protein identification thresholds were set for the Scaffold research: 3.0% FDR at the protein level, at least 2 minimal peptides, and 1.0% FDR at the peptide level.

Label-free quantification was performed using the ProgenesisQI (for proteomics) software V4.0 (Nonlinear Dynamics, UK) as described previously.^19^ The comparison was made between the two monospecies *A. actinomycetemcomitans* biofilm lysates (i.e., D7S wild type versus D7S *hns* mutant) or between different types of multispecies biofilm lysates (i.e., 6-species biofilm, 7-species biofilm containing D7S wild-type strain, and 7-species biofilm containing D7S *hns* mutant strain). Briefly, raw files of each individual run were imported to Progenesis and aligned with a pool of all samples as align reference. Then, peak picking was applied to the aligned feature using default settings from Progenesis for feature detection, alignment, and quantification. Up to 6 of the best-ranked ms/ms spectra per aligned peptide ion were exported into a mascot generic file (mgf) using the top 200 peaks and de-isotoping as well as charge deconvolution. Mascot results were loaded into Scaffold. Using filter options of min1 pep, 10% protFDR, and 5% pepFDR, the spectrum report was reimported into ProgenesisQI software. Only unique peptides were included for quantification. For identification, we used all proteins identified with at least two features. Proteins were grouped with ProgenesisQI and only non-conflicting features were used for quantification. For protein quantification, the normalized abundance of all non-conflicting peptide ions from the same protein group were summed together individually for each sample. This generates the normalized quantitative protein abundance. For statistical testing, the parametric test (analysis of variance) on the transformed (hyperbolic arcsine transformation) normalized protein abundance was applied. A heat map was used to obtain a global visualization and assessment of protein expression in different biofilms and to remove obvious outliers (Supplementary Fig. 3). The cluster analysis and heat maps were generated using the R programming language (R Core Team) and additional packages such as quantable and gplots (CRAN). As there were no outliers in the mono-species biofilms, all three replicates were taken under consideration for quantification. In contrast, there were a few samples in the multispecies biofilms could not be associated with any obvious factor and were considered as outliers and removed. The final number of biological replicates used for protein quantification included the following groups: D7S biofilm (*n* = 6), D7S *hns* biofilm (*n* = 8), and 6-species biofilm (*n* = 7). Quantified proteins with significant raw *p* value (*p* < 0.05), at least two unique peptides, and absolute value of log_2_ fold-change ≥1 were considered as true regulated proteins.

The mass spectrometry proteomics data have been deposited to the ProteomeXchange Consortium via the PRIDE^50^ partner repository with the dataset identifier PXD008444.

### Ontology analysis

GO terms from all regulated proteins for each comparison were put together to estimate the role of *A. actinomycetemcomitans* to entire biofilms or to their co-cultured species within the biofilm. The GO lists were generated with Uniprot (released on May 2017). For this, “Retrieve/ID Mapping” function was utilized with redundant terms removed based on analyses from REVIGO (released on May 2017) using the “small (0.5)” similarities. These GO lists were then manually summarized based on the defined classification of gene nomenclatures (molecular functional, biology process, and cellular component) on GO terms and presented in bar charts. The GO terms representing less than 2% of the whole GO in each domain were clustered into the category “other”.

### Statistical analysis

Unpaired *t*-test or a one-way ANOVA was used to calculate the statistical significances of the microbiological data from biofilm growth and CFU counting. For the latter, Bonferroni post hoc test was used for comparisons between the individual groups (Prism v.6 software GraphPad). The data were considered significant at *p* *<* 0.05. The results are presented as means ± standard deviations.

### Data availability

The mass spectrometry proteomics data have been deposited to the ProteomeXchange Consortium via the PRIDE partner repository with the dataset identifier PXD008444. The authors declare that all other data supporting the findings of this study are available within the article and its Supplementary Information, or upon request from the corresponding author.

## Electronic supplementary material

Supplementary figures

Supplementary Table 1

Supplementary Table 1

Supplementary Table 3

Supplementary Table 4

Supplementary Table 5

Supplementary Table 6
