# Supplementary material for: Aggregatibacter actinomycetemcomitans H-NS promotes biofilm formation and alters protein dynamics of other species within a polymicrobial oral biofilm
Source: NPJ Biofilms Microbiomes. 2018 May 22;4:12. doi: 10.1038/s41522-018-0055-4 (PMC5964231; doi:10.1038/s41522-018-0055-4)
Supplement: Supplementary file 1 — Supplementary figures [file 41522_2018_55_MOESM1_ESM.docx]

**
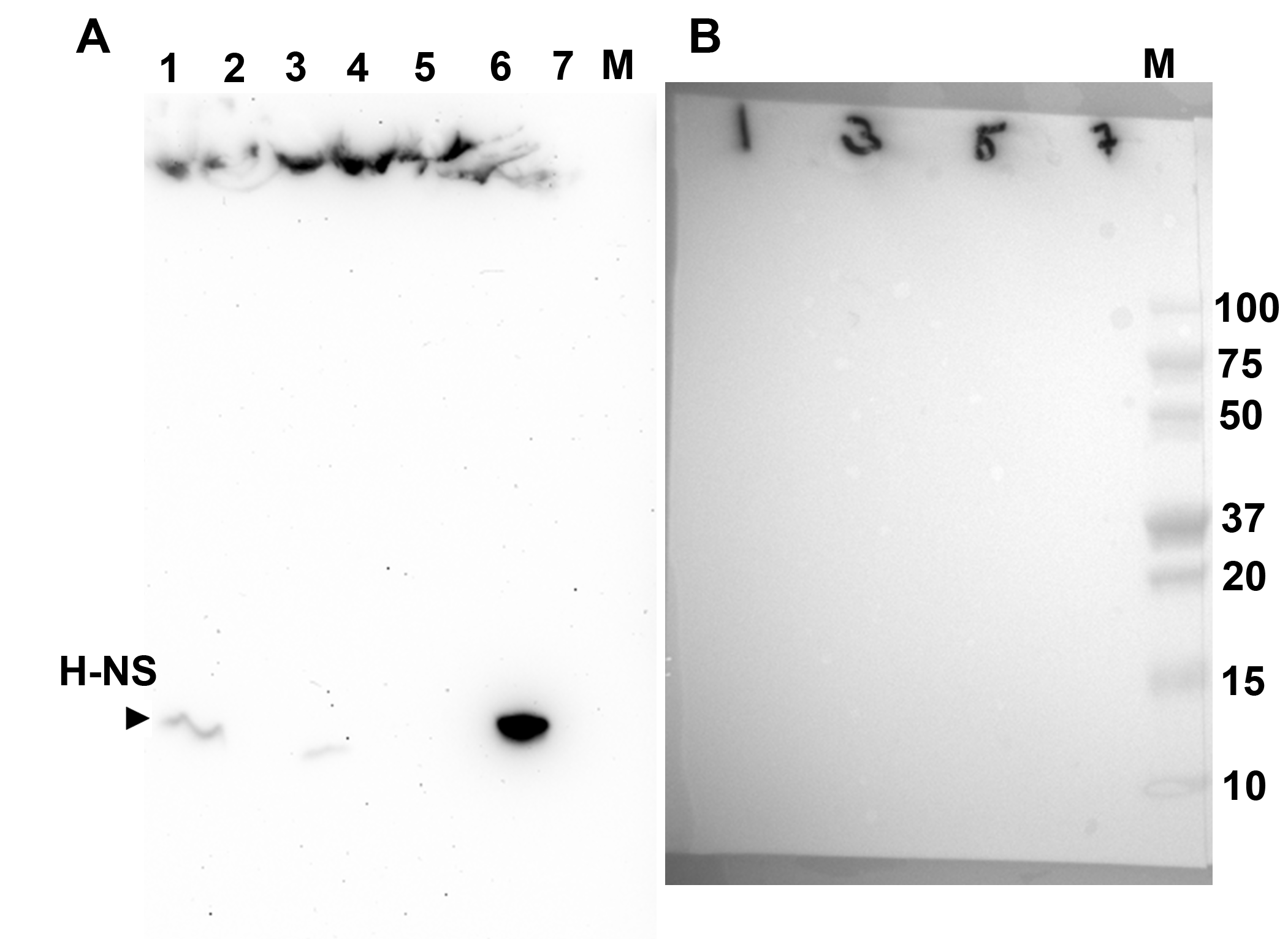
**

**Supplementary Figure 1. Detection of H-NS production in *A. actinomycetemcomitans*.** (A) Western blot using a polyclonal antiserum specific for *E. coli* H-NS (final dilution 1:10,000). Whole cell extracts equal to 10 μg protein were applied on the gel. Samples: 1. *A. actinomycetemcomitans* D7SS, 2. D7SS *hns*, 3. *A. actinomycetemcomitans* D7S, 4. D7S *hns* (clone 1), 5. D7S *hns* (clone 2), 6. *E. coli* JGJ102 (*hns*^+^; positive control), and 7. JGJ103 (*hns*; negative control). The reactive band corresponding to H-NS is indicated with an arrowhead. (B) The Western blot membrane analysed using epi-white illumination to visualize the proteins in the pre-stained molecular weight marker, which were loaded in lane M. Their sizes (kDa) are indicated along the right side. The detections shown in A and B were derived from the same experiment and processed in parallel.


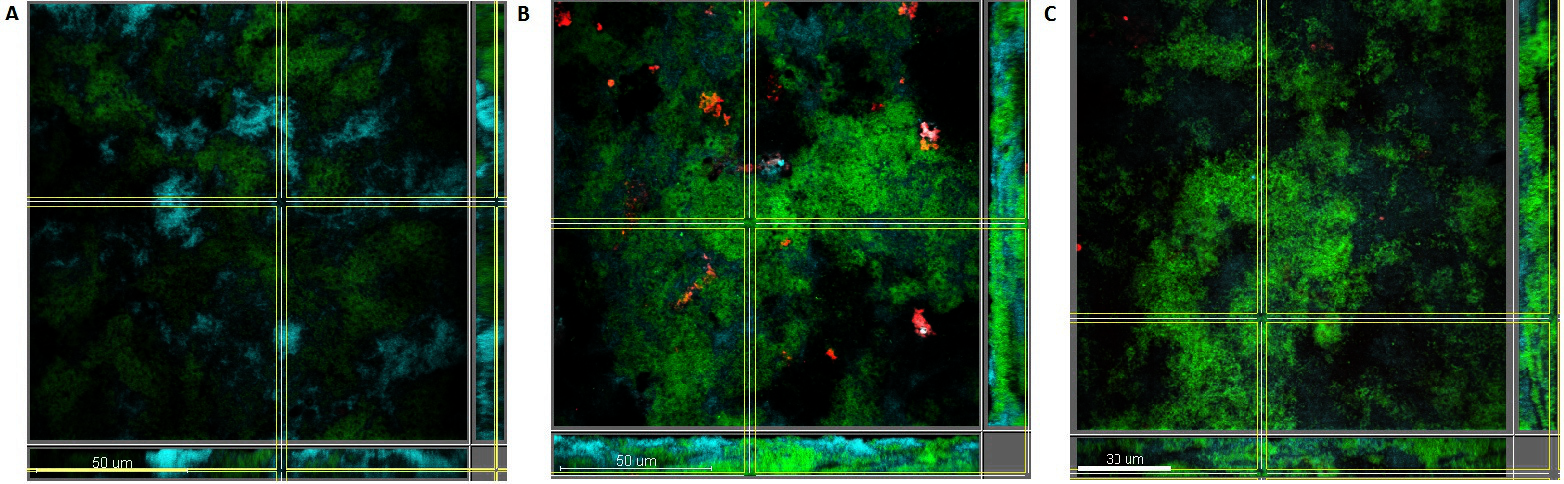


**Supplementary Figure 2. Representative confocal laser scanning microscopy images of multispecies biofilms.** Confocal laser scanning microscopy images of a typical (A) 6-species biofilm, (B) D7S biofilm, and (C) D7S *hns* biofilm. *A. actinomycetemcomitans*, *F. nucleatum*, and other species here appear red, blue and green, respectively. Scale bar: 50 µm.

**
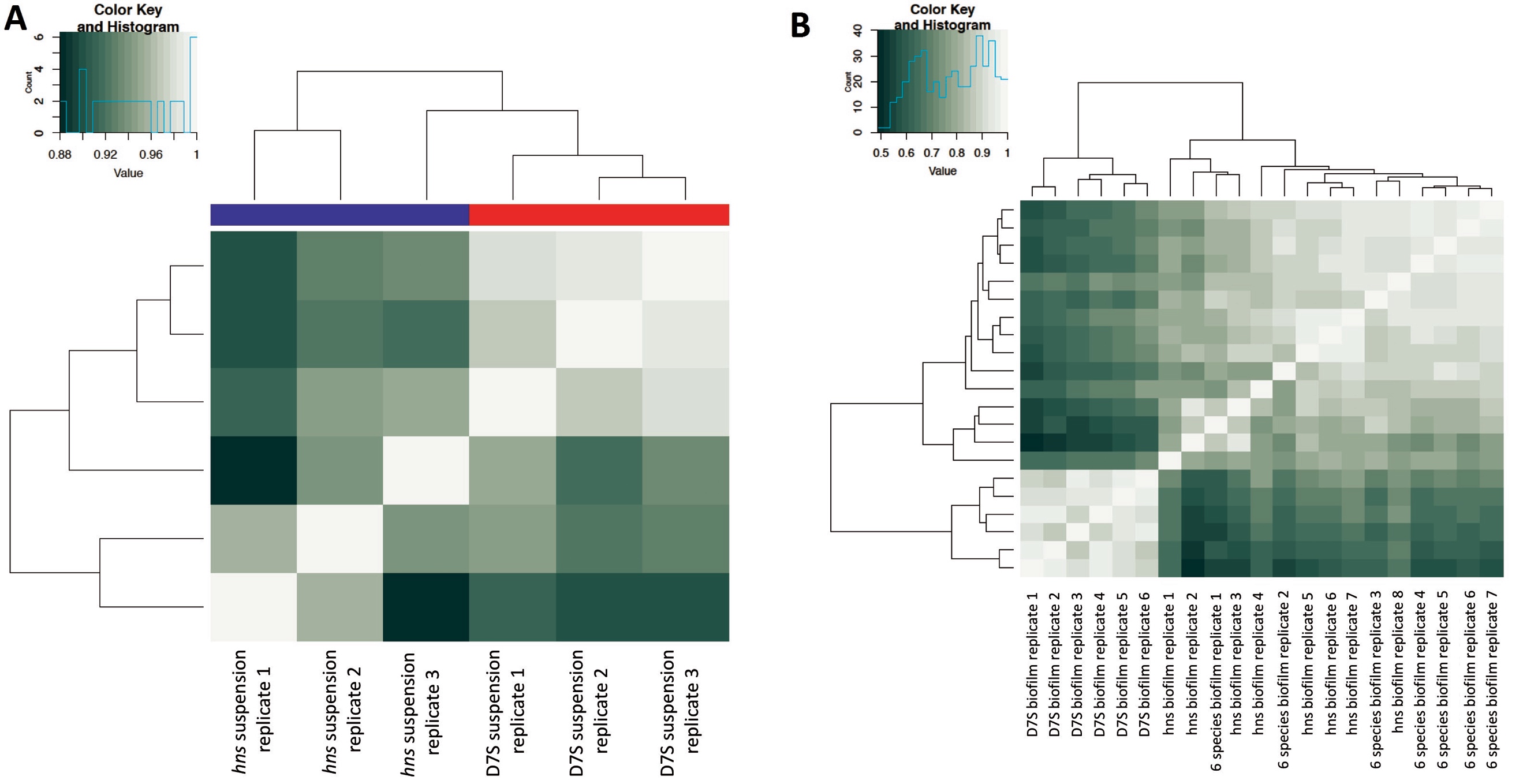
**

**Supplementary Figure 3. Quality control of the label-free quantitation data.** Heatmaps of integrated peptide feature intensities were displayed to compare the protein profiles obtained from the *A. actinomycetemcomitans* monocultures on agar (A), and the different biofilms (B).
